# Relapse of cryoglobulinemic vasculitis with new-onset severe renal involvement in two patients following mRNA COVID-19 vaccination: A case report

**DOI:** 10.1097/MD.0000000000029431

**Published:** 2022-06-10

**Authors:** Alexandra Vornicu, Andreea Berechet, Georgiana Frățilă, Bogdan Obrişcă, Ciprian Jurcuţ, Gener Ismail

**Affiliations:** a Department of Nephrology, Fundeni Clinical Institute, Bucharest, Romania; b “Carol Davila” University of Medicine and Pharmacy, Bucharest, Romania; c Department of Internal Medicine, “Dr. Carol Davila” Central University Emergency Military Hospital, Bucharest, Romania.

**Keywords:** case report, COVID-19, mRNA vaccination, vasculitis, cryoglobulinemic vasculitis

## Abstract

**Rationale:**

Since mass-scale severe acute respiratory syndrome coronavirus 2 vaccination, there have been case reports of several immune-mediated reactions, including new-onset and flares of glomerular disorders following immunization with mRNA coronavirus disease 2019 vaccines. Here, we report two cases, the first to our knowledge, of relapsing cryoglobulinemic vasculitis with new-onset severe renal involvement following mRNA coronavirus disease 2019 vaccination.

**Patient concerns:**

The relapse of the cutaneous and the new onset of severe renal involvement of cryoglobulinemic vasculitis occurred three weeks after the second dose of the mRNA Moderna coronavirus disease 2019 vaccination and two days after the first dose of mRNA Pfizer coronavirus disease 2019 vaccination in the first and second patient, respectively.

**Diagnosis:**

Kidney biopsies were performed. The first pacient's kidney biopsy showed a membranoproliferative pattern of glomerular injury with extensive mesangial and endocapillary hypercellularity, while severe endothelial swelling, loss of fenestrations and widening of subendothelial space were identified by electron-microscopy. The second patient's kidney biopsy was consistent with cryoglobulin associated membrano-proliferative pattern of glomerular injury.

**Interventions:**

Our patients were managed with a combination of immunosuppressants consisting of corticosteroids, Cyclophosphamide and Rituximab with a favourable outcome at the end of the induction period.

**Outcomes:**

Clinical and immunological response was achieved in both patients after four months of follow-up.

**Lessons:**

The temporal association of the relapse of the cryoglobulinemic vasculitis to mRNA coronavirus disease 2019 vaccination suggest that the vaccine might have been a trigger for the reactivation of the disease in our cases. This possible association should be acknowledged by physicians in order to provide optimal monitoring and treatment in case of reactivation of the disease post-immunization.

## 1 Introduction

The rapid and mass severe acute respiratory syndrome coronavirus 2 (SARS-CoV-2) vaccination has provided effective protection against severe forms of coronavirus disease 2019 (COVID-19) infection,^[1]^ but has raised new concerns about the occurrence of several immune-mediated reactions, including new-onset and flares of glomerular disorders(mainly IgA nephropathy, ANCA-associated vasculitis, minimal change-disease or membranous nephropathy).^[2,3]^ Here, we report two cases, the first to our knowledge, of relapsingcryoglobulinemic vasculitis with new-onset severe renal involvement following mRNA COVID-19 vaccination.

## 2 Case presentation

### 2.1 Case 1

A 51-year-old female patient with a past medical history of primary Sjögren syndrome (diagnosis based on 2016 ACR-EULAR Classification Criteria for primary Sjogren's Syndrome^[4]^) and cryoglobulinemic vasculitis with cutaneous and nervous involvement (diagnosis based on 2011 Preliminary classification criteria for the cryoglobulinaemic vasculitis^[5]^), treated in the last two years with oral corticosteroids and hydroxychloroquine, received the second dose of the mRNA Moderna COVID-19 vaccination in March 2021. After three weeks she developed a reactivation of the cutaneous vasculitis with palpable purpura and ulcers and presented with worsening bilateral lower extremity pitting edema. On admission, the laboratory assessment showed acute kidney injury and nephrotic syndrome with active urinary sediment (Table 1). She had no prior history of SARS-CoV2 infection or renal involvement secondary to Sjogren syndrome or cryoglobulinemic vasculitis. The HCV serology was negative and the cryoglobulins were composed of polyclonal immunoglobulins.

**Table 1:** Laboratory investigations at cryoglobulinemic vasculitis relapse and after 4 months of follow-up.

	Case 1		Case 2		
Laboratory parameters	Baseline	After 4 mo	Baseline	After 4 mo	Reference ranges
Serum creatinine (mg/dl)	2.02	1.06	2.09	1.37	0.5–1.02
Serum urea (mg/dl)	116	58	89	44	12–45
Serum sodium (mmol/l)	130	139	141	143	136–145
Serum potassium (mmol/l)	4.5	4.36	4.39	4.12	3.5–5.1
Serum albumin (g/dl)	2.95	4.01	3.76	4.65	3.4–5
Proteinuria/24 h (g/d)	4	0.8	1.2	0.2	<0.14
Hematuria (RBC/mmc)	242	25	50	20	0–30.7
Hemoglobin (g/dl)	7.5	12.7	10.5	12.7	11.5–17
ANA (U/ml)	4.9	4	6	8.8	0–1.1
Anti-SS-A (U/ml)	114	71.5	82.9	43	0–25
Anti-SS-B (U/ml)	32	19.5	0.1	0.1	0–25
Rheumatoid factor (IU/ml)	163	9.38	85.3	13	0–15
C3 (mg/dl)	50	126	66	116	90–180
C4 (mg/dl)	2	12	3	20	10–40
C-reactive protein (mg/l)	109	3	22	2.4	0–3

**Figure 1:**
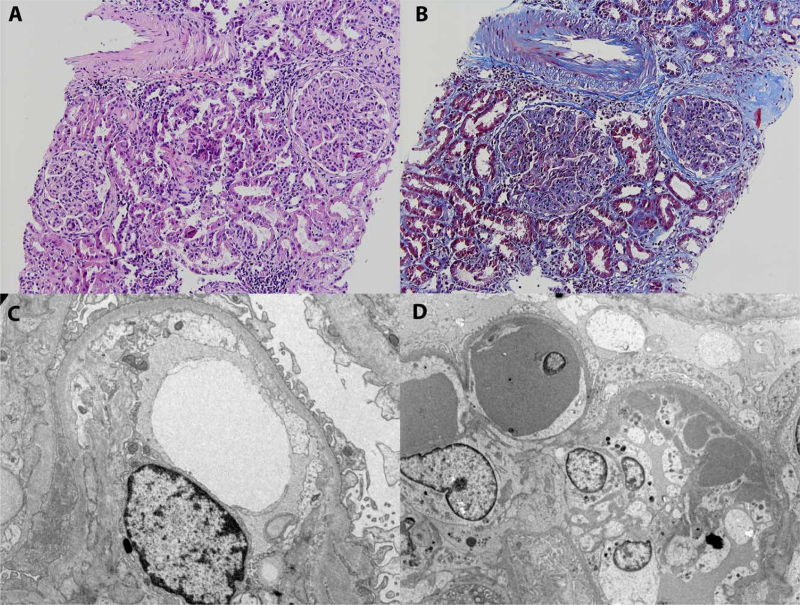
Case 1 (A, C). Light microscopy, hematoxylin and eosin (A) staining. Membrano-proliferative pattern of glomerular injury with mesangial and endocapillary hypercellularity. Electron microscopy (C). Severe lesions of thrombotic microangiopathy with diffuse endothelial injury, loss of fenestrations and widening of subendothelial space. In addition, extensive foot process effacement was noted. Case 2 (B, D). Light microscopy, trichrome masson (B) staining.Membrano-proliferative pattern of glomerular injury with cryoglobulin plugs, mesangial and endocapillary hypercellularity. Electron microscopy (D). Extensive subendothelial electron-dense deposits and intraluminal cryoglobulin plugs with microtubular substructure on higher magnification.

The kidney biopsy showed a membranoproliferative pattern of glomerular injury with extensive mesangial and endocapillary hypercellularity, while severe endothelial swelling, loss of fenestrations and widening of subendothelial space were identified by electron-microscopy (Fig. 1).

**Figure 2:**
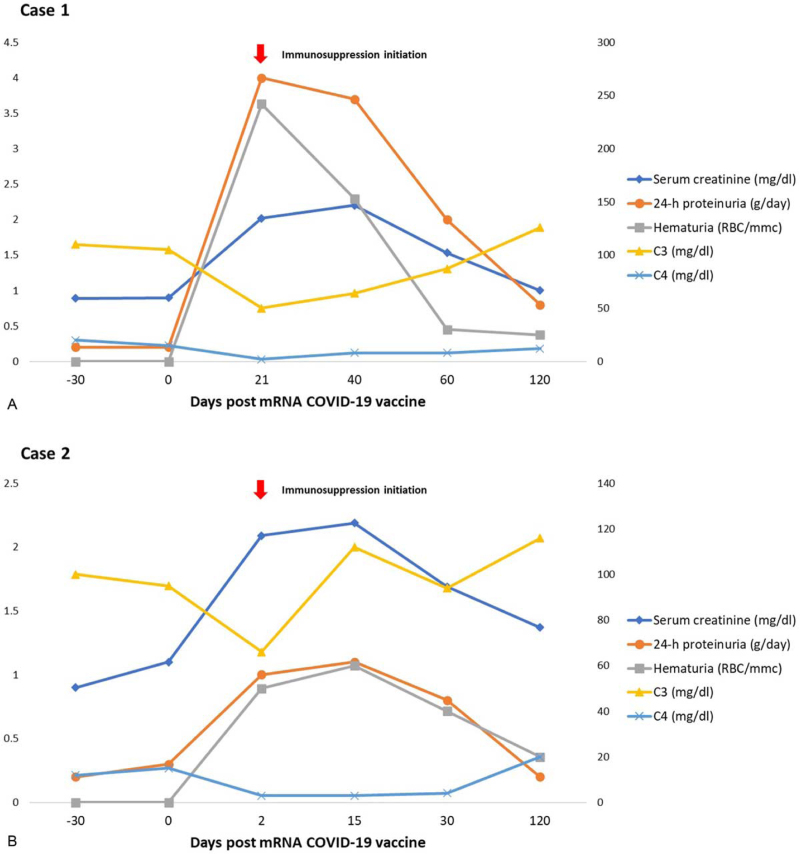
Patient's evolution post-mRNA COVID-19 vaccination.

The patient was started on an induction immunosuppressive treatment regimen consisting of a combination of intravenous Cyclophosphamide, Rituximab and corticosteroids. After four months, the patient achieved a normalization of renal function, a resolution of the hematuria and significant improvement of proteinuria (below 1 g/d) paralleled by a complete immunological remission (normalization of rheumatoid factor and complement level, resolution of the inflammatory syndrome) (Table 1, Fig. 2). The patient presented complete lymphocyte B depletion after four months of treatment.

### 2.2 Case 2

A 59-year-old female patient with a past medical history of primary Sjögren syndrome(diagnosis based on 2016 ACR-EULAR Classification Criteria for primary Sjogren's Syndrome^[4]^) for nine years with cryoglobulinemic vasculitis with cutaneous and nervous involvement (diagnosis based on 2011 Preliminary classification criteria for the cryoglobulinaemic vasculitis^[5]^) since January 2021, for which she was started on hydroxychloroquine and oral corticosteroids, received the first dose of the mRNA Pfizer COVID-19 vaccination in March 2021. After 2 days she presented with fatigue, fever, myalgias, palpable purpura and small cutaneous malleolar ulcers. On admission, the laboratory assessment revealed acute kidney injury and acute nephritic syndrome (Table 1). She had no prior history of SARS-CoV-2 infection or renal involvement secondary to Sjogren syndrome or cryoglobulinemic vasculitis. The HCV serology was negative and the cryoglobulins were composed of polyclonal immunoglobulins.

The kidney biopsy was consistent with cryoglobulin associated membrano-proliferative pattern of glomerular injury (Fig. 1).

The patient was started on an induction immunosuppressive treatment regimen consisting of a combination of intravenous Cyclophosphamide, Rituximab and corticosteroids. After four months, the patient achieved complete clinical remission (improvement in kidney function with a serum creatinine of 1.3 mg/dl, no urinary abnormalities, no active cutaneous lesions) and complete immunological remission (rheumatoid factor of 13 U/ml, complement factors within normal ranges, without inflammatory syndrome). The patient presented complete lymphocyte B depletion after one month of follow-up.

## 3 Discussion

Several cases of new-onset vasculitis following vaccination have been reported in the past,^[6]^ including one case report of de novo type II mixed cryoglobulinemia following influenza and pneumococcal immunization.^[7]^ Since mass-scale SARS-CoV-2 vaccination, there have been case reports of new-onset anti-GBM and ANCA-associated vasculitis following immunization with mRNA COVID-19 vaccines.^[3]^ We have described, the first to our knowledge, two cases of relapsing cryoglobulinemic vasculitis with new-onset severe renal involvement following mRNA COVID-19 vaccination.

Immune-mediated reactions seem to occur in a small proportion of COVID-19 vaccinated people, but the true incidence of new onset or relapse of autoantibody-mediated kidney disease following COVID-19 immunization is difficult to establish.^[2,3]^ IgA nephropathy is the most frequent reported glomerulonephritis post-COVID-19 vaccination in the literature.^[3]^ Klomjit et al reported the largest case series (n = 13) of de novo and relapsing glomerulonephritis post-COVID-19 vaccination and observed that most patients (77%) presented after the second dose with a median time of onset ranging from one to six weeks.^[3]^ Moreover, 38% of these patients had a previously diagnosed autoimmune disease.^[3]^ Both our patients had a previous diagnosis of Sjögren syndrome and cryoglobulinemic vasculitis with cutaneous and nervous involvement, both being in clinical remission prior to COVID-19 vaccination, on treatment with oral corticosteroids and hydroxychloroquine; the treatment was not stopped or reduced prior or post-vaccination. The relapse of the cutaneous and the new onset of severe renal involvement of cryoglobulinemic vasculitis occurred three weeks after the second dose of the mRNA Moderna COVID-19 vaccination and two days after the first dose of mRNA Pfizer COVID-19 vaccination in the first and second patient, respectively. Moreover, in both patients, the cryocrit was <2% before and >2% after vaccination. The underlying immune dysregulation of these patients might have predisposed them to the development of the relapse of cryoglobulinemic vasculitis with severe renal involvement. The risk of developing a flare after COVID-19 vaccination in the patients with systemic rheumatic diseases seem to be low.^[8,9]^ An international survey that included 2860 patients with systemic rheumatic disease, revealed a 4.6% rate of flares that required medication changes.^[8]^ Similarly, in the VACOLUP survey, 21 (3%) of the 696 systemic lupus erythematosus patients included, developed a flare after COVID-19 vaccination, two of them with renal involvement.^[9]^

Several studies and the real-world data based on people using the mRNA-vaccines seems to conclude that this technology is superior in terms of the potency of the immune responses over inactivated vaccine and even natural infection by inducing robust antigen specific T-cell and prolonged germinal center B-cell responses.^[2,3]^ The temporal association between mRNA COVID-19 vaccination and the induction of autoantibody-mediated glomerular disease may suggest a potential pathogenic link that can be explained through several potential mechanisms, like molecular mimicry or bystander activation, although the pathogenesis has not been yet fully elucidated.^[2]^

Our patients were managed with a combination of immunosuppressants consisting of corticosteroids, Cyclophosphamide and Rituximab with a favourable outcome at the end of the induction period. Clinical and immunological response was achieved in both patients after four months of follow-up. The outcome was frequently favourable in the reported cases of new-onset or relapse of glomerulonephritis post-COVID19 immunization, some having spontaneous resolution, others achieving partial or complete remission using conventional therapeutic options for these diseases.^[2,3,10]^

The provisional recommendations for SARS-CoV-2 vaccination recently published by the Task Force of the Italian Group for the Study of Cryoglobulinemia propose that those patients with cryoglobulinemic vasculitis to be vaccinated with priority, ideally with mRNA vaccines.^[11]^ They addressed also the risk of reactivation of the disease associated with immunization, but conclude that the benefits seem to outweigh the risks, taking into account the available data.^[11]^

In conclusion, the temporal association of the relapse of the cryoglobulinemic vasculitis to mRNA COVID-19 vaccination suggest that the vaccine might have been a trigger for the reactivation of the disease in our cases. In our patients, COVID-19 mRNA vaccination triggered a clinical phenotype switch with development of new-onset, severe, renal involvement. This association should be acknowledged by physicians in order to provide optimal monitoring and treatment in case of reactivation of the disease post-immunization.

## Author contributions

**Conceptualization:** Alexandra Vornicu, Bogdan Obrisca, Gener Ismail

**Data curation:** Alexandra Vornicu, Bogdan Obrisca

**Supervision** Ciprian Jurcut and Gener Ismail. All authors have read and agreed to the published version of the manuscript

**Writing – original draft:** Alexandra Vornicu, Andreea Berechet, Georgiana Fratila, Bogdan Obrisca, Gener Ismail

**Writing – review & editing:** Alexandra Vornicu, Andreea Berechet, Bogdan Obrisca, Georgiana Fratila, Gener Ismail
